# Novel Electrospun Gelatin Nanofibers Loaded with Purple Potato Anthocyanin and Syringic Acid for Multifunctional Food Packaging

**DOI:** 10.3390/foods13162538

**Published:** 2024-08-14

**Authors:** Chen Huang, Junjie Tang, Xingyan Chen, Xinxin Zeng, Weiquan Zhong, Jie Pang, Chunhua Wu

**Affiliations:** College of Food Science, Fujian Agriculture and Forestry University, Fuzhou 350002, China; hc05272000@163.com (C.H.); junjietang0907@163.com (J.T.); 18950293503@163.com (X.C.); 17759866181@163.com (X.Z.); z18296705390@163.com (W.Z.); pang3721941@163.com (J.P.)

**Keywords:** gelatin nanofibers, anthocyanin, syringic acid, multifunctional packaging

## Abstract

In this study, a series of novel nanofibers based on gelatin (GA) loading with purple potato anthocyanin (PPA) and syringic acid (SA) were obtained by electrospinning technology. The effects of SA on mechanical properties, thermal stability, antioxidant capacity, and antimicrobial activity of the GA/PPA nanofibers were systematically characterized. The scanning electron microscopy observation results revealed a smooth surface on the nanofibers. The incorporation of SA enhanced the viscosity of the electrospun solutions, and it increased the average diameter of nanofibers from 0.17 μm to 0.28 μm. The tensile strength and thermal stability of the obtained nanofibers were enhanced with the addition of a suitable level of SA (1.5%, *w*/*v*), which strengthened the intermolecular interaction. The GA/PPA/SA nanofibers presented over 80% antioxidant capacity and strong antibacterial activity against *E. coli* and *S. aureus*. Meanwhile, the sensitivity responses of nanofibers to NH_3_ revealed that GA/PPA/SA II nanofibers (1.5% *w*/*v* SA) presented good sensitivity of colorimetric behavior to ammonia. A pork spoilage test was performed to evaluate practical application of the nanofibers, and an obvious color change (dark purple to green) was observed. These results indicated GA/PPA/SA II nanofibers can be utilized as an active and intelligent multipurpose packaging material to preserve and track the freshness of pork.

## 1. Introduction

There is a growing interest in developing biopolymer-based active and intelligent food packaging film [1]. Advanced active and smart packages allow consumers to check food quality through visual colorimetric changes in addition to their protective functions. These multifunctional films are designed to monitor the freshness of food products in real time during transportation and sale processes [2,3]. High-protein-content foods that have begun to deteriorate may emit volatile alkaline substances, such as ammonia, which can lead to pH changes [4]. These changes can affect the color change of pH response pigments. Therefore, the incorporation of pH response pigments in food packaging is a useful method to indicate the freshness of food through color changes [5]. Natural pigment-developing films have gained more and more interest due to their good biocompatibility and pH response [6,7,8]. One natural pigment that has gained significant popularity in the creation of intelligent films is the lipophilic pigment anthocyanin, extracted from purple potatoes. This pigment is known for its intense color variation response to pH changes and its associated health-promoting benefits [9].

Among food-grade biopolymers, gelatin (GA) is a cost-effective natural protein derived from the partial hydrolysis of collagen. It is considered to be non-immunogenic, biodegradable, simple to process, and well biocompatible [10,11]. Nevertheless, pure GA films have insufficient functional characteristics (e.g., antioxidant capacity and antimicrobial activities) required for active packaging. Natural plant phenolic acids such as syringic acid (SA), gallic, and trans-cinnamic acids have been added into GA-based material to improve the antioxidant capacity and antimicrobial activity of packaging film [12,13]. SA is a phenolic acid compound commonly found in fruits, vegetables, and cereals. It is synthesized in plants through the shikimic acid pathway and possesses antioxidant, antibacterial, anti-inflammatory, anti-endotoxin, and other biological activities [14,15]. The various activities of SA depend mainly on the presence of hydroxyl (-OH) and methoxy (-OCH_3_) groups in the aromatic ring. Yang et al. [16] utilized SA to enhance the antibacterial and antioxidant capacity of chitosan films.

Electrospinning is an easy, multipurpose, and economical technology for obtaining nano-scale fibers from polymers [17,18]. Electrospun nanofibers possess several distinct features including high porosity, high surface-to-volume ratio, nanoporous structure, and high absorbance properties that set them apart from conventional casting films [19,20]. In reported studies, Kasitnun et al. used electrospinning technology to prepare indicator membranes to detect food freshness [7], and Dai et al. developed a novel nanofibrous film with antibacterial, antioxidant, and thermoregulatory functions fabricated by coaxial electrospinning [21]. Wang et al. studied dual-functional intelligent gelatin-based packaging film for maintaining and monitoring shrimp freshness [22]. Goudarzi et al. developed fiber mats to monitor the freshness and enhance the shelf-life quality of minced beef meat [23]. The results of previous studies show the advantages of nanofibers that are eco-friendly, low-cost, and bacteriostatic [7,21,22,23]. Some studies have used coaxial electrospinning methods; the electrospinning method in this study was simpler and initially explored the effect of SA on the pigment response to ammonia. To the best of our knowledge, the utilization of electrospun GA nanofibers containing PPA and SA as active and intelligent food packaging film has not been explored.

Therefore, the major aim of this work was to prepare electrospun GA nanofibers incorporated with PPA and SA via an electrospinning technique. The effect of the SA concentrations on morphology, thermal stability, antioxidant capacity, and antibacterial activity of GA/PPA electrospun nanofibers were evaluated. Additionally, the responsiveness of electrospun nanofibers to ammonia was also investigated. Subsequently, the GA/PPA/SA II film, which exhibited the strongest mechanical property and the highest sensitivity to ammonia, was also applied to monitor the freshness of pork fillet. Physical properties, pH sensitivity, volatile ammonia sensitivity, antioxidant, and antibacterial properties of the nanofilm were characterized, as well as practical application pertaining to the preservation and monitoring of pork freshness.

## 2. Materials and Methods

### 2.1. Materials

Gelatin (type B, m_w_~100 kDa) and syringic acid (purity ≥ 98%) were purchased in Mecklin Biochemical Co., Ltd. (Shanghai, China). Fresh pork fillet was obtained from Yong Hui supermarket (Fuzhou, China), and purple potato anthocyanin from Xi’an Huilin Biological Technology Co., Ltd. (Xi’an, China). In this study, other reagents used were analytical grade.

### 2.2. UV–vis Spectroscopy Measurement 

The UV–vis spectra of PPA in a series of phosphate buffer solutions (pH ranging from 2 to 12) were measured by UV-2600 spectrophotometer (Shimadzu, Japan) with a scanning wavelength range of 400–700 nm at a test temperature of 25 °C. The color changes of the PPA solution at different pH levels were also recorded using a camera. 

### 2.3. Preparation of Nanofibers 

A certain amount of PPA power was fully dissolved in acetic acid solution (60% *v*/*v*) to obtain 4% (*w*/*v*) PPA solution. Different concentrations of SA were added to the PPA solution sequentially and mechanically stirred for 30 min to obtain the GA/PPAGA/PPA/SA solution. Next, 7.5 g of gelatin powder was slowly added into the PPA/SA solution and stirred at 25 °C for 12 h, followed by standing for another 12 h. This process resulted in the formation of different spinning solutions: the GA/PPA solution, GA/PPA/SA I solution (1% *w*/*v* SA), GA/PPA/SA II solution (1.5% *w*/*v* SA), and GA/PPA/SA III (2% *w*/*v* SA) solution. Subsequently, GA/PPA nanofibers, GA/PPA/SA I nanofibers, GA/PPA/SA II nanofibers, and GA/PPA/SA III nanofibers were obtained by electrospinning. The main properties of the electrospun solutions are shown in Table 1. The electrospinning equipment parameters used in this study included a receiving distance of 15 cm, set voltage of 18 kV, and jet speed of 0.1 mm/min.

### 2.4. Characterizations

#### 2.4.1. The Viscosity of Electrospun Solutions

The viscosity of the electrospun solutions was analyzed using a rheometer (Anton Paar, Graz, Austria) with a pillar and plate geometry (PP50-SN23616). The rheometer parameters used in this study were a gap distance of 1 mm and shearing rates within a range of 0.1 s^−1^ to 100 s^−1^. The tests were conducted three times at a temperature of 25 °C, and the average values were obtained.

#### 2.4.2. Scanning Electron Microscopy (SEM)

The micromorphology of the GA/PPA and GA/PPA/SA nanofibers was measured by scanning electron microscope (TESCAN MIRA LMS, Tokyo, Japan) with a magnification of 10,000. The diameter distribution of the nanofiber samples was measured using ImageJ software.

#### 2.4.3. Fourier Transform Infrared (FT-IR) Spectroscopy 

Nanofiber samples were mixed with the potassium bromide powders at a weight ratio of 1:100, followed by mechanical compression into slices. The obtained samples were scanned in the range of 400–4000 cm^−1^ on an FT-IR spectrometer (Thermo Fisher Scientific Co., Ltd., Waltham, MA, USA) with a resolution of 4 cm^−1^ at room temperature.

#### 2.4.4. X-ray Diffraction (XRD) Spectra 

The XRD spectra of GA/PPA, GA/PPA/SA I, GA/PPA/SA II, and GA/PPA/SA III nanofibers were analyzed by X-ray diffractometer (Bruker Inc., Karlsruher, Germany) with Cu Kα radiation in the scattering range of 2θ (5–70°) with steps of 2° (2θ)/min. 

#### 2.4.5. Thermogravimetric Analysis (TGA)

The thermal stability of the nanofibers was measured under a nitrogen atmosphere by thermo-gravimetric analyzer STA409-PC (Netzsch, Selb, Germany) from 30 to 600 °C at a heating rate of 10 °C/min, with a nitrogen gas flow rate of 50 mL/min. Each nanofiber sample of around 5.0 mg was accurately weighed.

#### 2.4.6. Mechanical Properties 

An AG-IC50 kN Texture Analyzer (Shimadzu, Tokyo, Japan) was utilized to measure the mechanical properties of the nanofiber samples. The tensile speed parameter was set at 5 mm/min. All nanofiber samples were tested 5 times in parallel to obtain accurate data.

#### 2.4.7. Antioxidant Test 

The antioxidant activity of the nanofiber samples was assessed via the DPPH method. A specific amount of nanofiber sample (0.05 g) was fully dissolved in 10 mL DPPH solution (40 mg/L). The absorbance value was tested at the wavelength of 517 nm using a UV-2600 spectrophotometer (Shimadzu, Tokyo, Japan), after 30 min. The measurement was performed 3 times. The following formula was used to calculate the free-radical scavenging rate:

$$Radical\;scavenging\;rate\left(\%\right)=\frac{A_{0}-A_{1}}{A_{1}}\times 100\%$$

where *A*_0_ and *A*_1_ represent the absorbance values of the control group and sample group, separately. 

#### 2.4.8. Antibacterial Properties

In the antibacterial properties test, the agar disk diffusion method was selected to assess the antibacterial activity of the nanofibers against *S. aureus* and *E. coli* bacteria. Samples with a diameter of 8 mm were disinfected with ultraviolet light for 30 min to avoid bacterial contamination. Then, 100 μL of activated bacterial diluted solution was evenly coated on a Luria–Bertani agar plate. The ultraviolet light-treated samples were placed on the obtained Luria–Bertani agar plate and incubated at 37 °C for culturing for 24 h, and the diameter of the inhibition zone was measured for every sample.

#### 2.4.9. Sensitivity of Nanofibers to Ammonia

The ammonia-sensitivity of nanofibers was evaluated according to Yao et al.’s method [24], attaching nanofiber samples which were cut into square shapes (2 cm × 2 cm) onto the headspace of a sealed plate with 20 mL of 0.2 mol/L ammonia aqueous solution. The color changes of the nanofiber samples were recorded every 10 min by colorimeter (CHNSpec Technology Co., Ltd., Hangzhou, China).

#### 2.4.10. Application of Nanofibers in Monitoring the Quality Changes of Pork 

The pork spoilage trial was evaluated according to Ran et al.’s method [25]; 250 g fresh pork fillet was held in a sealed container, and the square-shaped (2 cm × 2 cm) nanofiber samples were attached to the tops of the containers and stored at 25 °C in an incubator for 4 days. The ΔE of every nanofiber sample was measured by colorimeter (CHNSpec Technology Co., Ltd., Hangzhou, China), and the total volatile basic nitrogen values of the pork were determined every day, using a Kjeldahl nitrogen apparatus (Foss, Danmark, German).

### 2.5. Statistical Analysis

All tests were conducted in triplicate and the data represented numerically, expressed as a mean ± standard deviation (SD). IBM SPSS software (SPSS Inc., Chicago, IL, USA) was used to compare variance (ANOVA), via Duncan’s similarity test, to verify the significance of all samples, and drawing was carried out in Origin 2021 software. In figures and tables, distinct letters indicate values with significant differences, whereas double letters indicate partial differences with intermediate similarities between the indicated letters. Data were defined as statistically significantly different if *p* < 0.05.

## 3. Results and Discussion

### 3.1. UV-vis Spectra and Color Variation of PPA in Different Buffer Solutions

**Figure 1:**
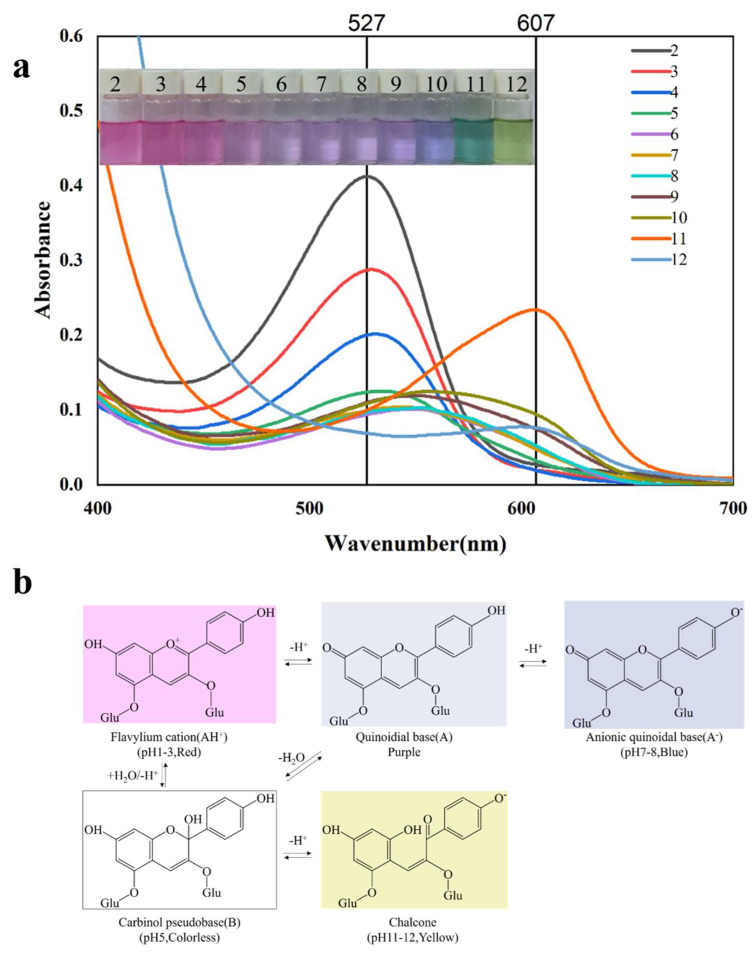
UV−vis spectra and color transformation of PPA solutions at different pH values (**a**), and corresponding structures of PPA at different pH values (**b**).

Obvious color changes were observed in PPA solutions across a pH range of 2 to 12, as represented in Figure 1a. At pH values of 2–4, the PPA solution appeared pink and gradually faded at pH 5. The solution color then transitioned to purple and deepened at the pH range of 7 to 10, eventually turning yellow at higher pH values of 11–12. In the PPA solution, there was a maximum absorption peak of 529 nm at pH 2, which shifted to 600 nm as the pH increased. In general, the structure of anthocyanins changed along with the pH: red flavylium cation at pH 1–3, carbinol pseudo base at pH5, anionic quinoidal base at pH 7–8, and chalcone pH 11–12 (Figure 1b) [26]. Therefore, the bathochromic shifts in the maximum absorption peak and observed color change are primarily put down to the different structures of anthocyanins at corresponding pH values. Similar phenomena have been found in rose anthocyanins, red radish anthocyanins, and black bean seed anthocyanins [3,14,20]. 

### 3.2. The Viscosity of Electrospun Solution

**Figure 2:**
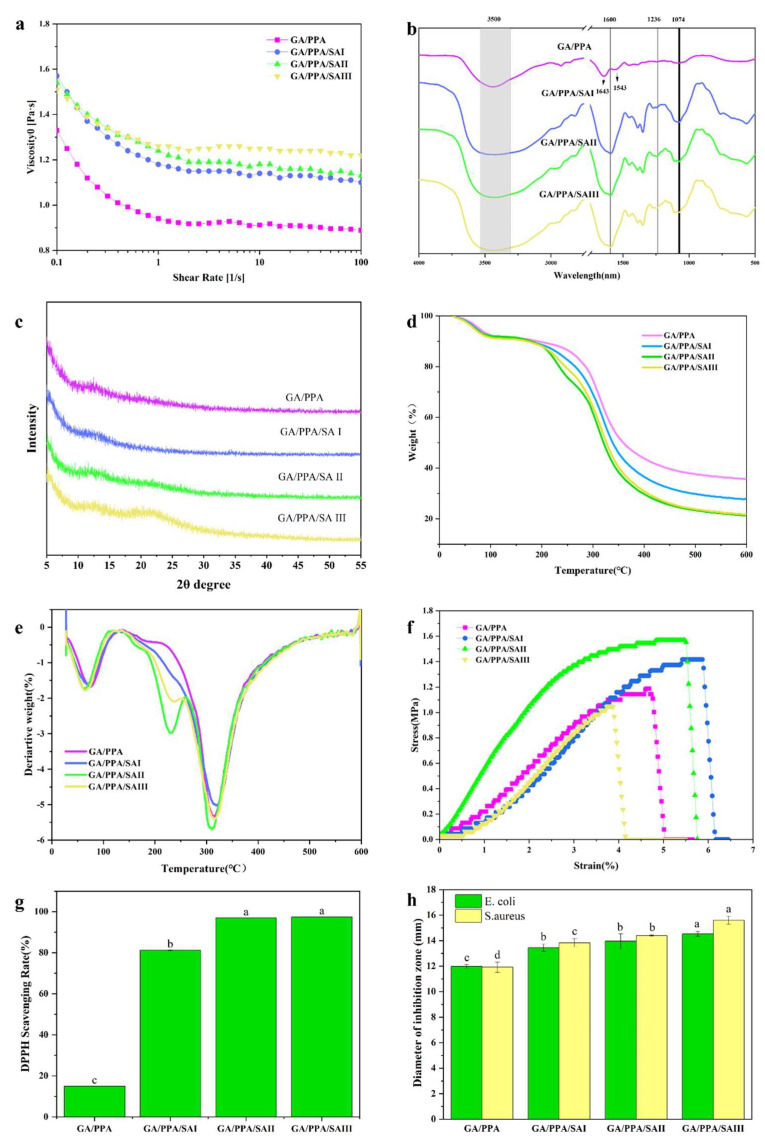
The viscosity of electrospun GA/PPA and GA/PPA/SA solution (**a**), FT-IR spectra of the GA/PPA and GA/PPA/SA nanofibers (**b**), XRD patterns of the GA/PPA and GA/PPA/SA nanofibers (**c**), TGA curves of the GA/PPA and GA/PPA/SA nanofibers (**d**), DTG curves of the GA/PPA and GA/PPA/SA nanofibers (**e**), strain–stress curve of the GA/PPA and GA/PPA/SA nanofibers (**f**), DPPH scavenging rate of GA/PPA and GA/PPA/SA nanofibers (**g**), and the antimicrobial activity of GA/PPA and GA/PPA/SA nanofibers (**h**). In (**g**,**h**), different letters indicate significant difference (*p* < 0.05).

Fiber morphology of the electrospun nanofibers was greatly affected by the viscosity of the electrospun solution. With the increase in solution viscosity, the diameter of gelatin fiber also increased significantly [27]. The viscosity of the electrospun solution varied with the concentration of SA at high shear rates, as illustrated in Figure 2a. It can be observed that with the addition of SA, the viscosity slightly increased accordingly; the reason for this could have been the increase in internal friction resistance of the electrospun solution and the decrease in molecular fluidity [28]. 

### 3.3. Morphology of Nanofibers

**Figure 3:**
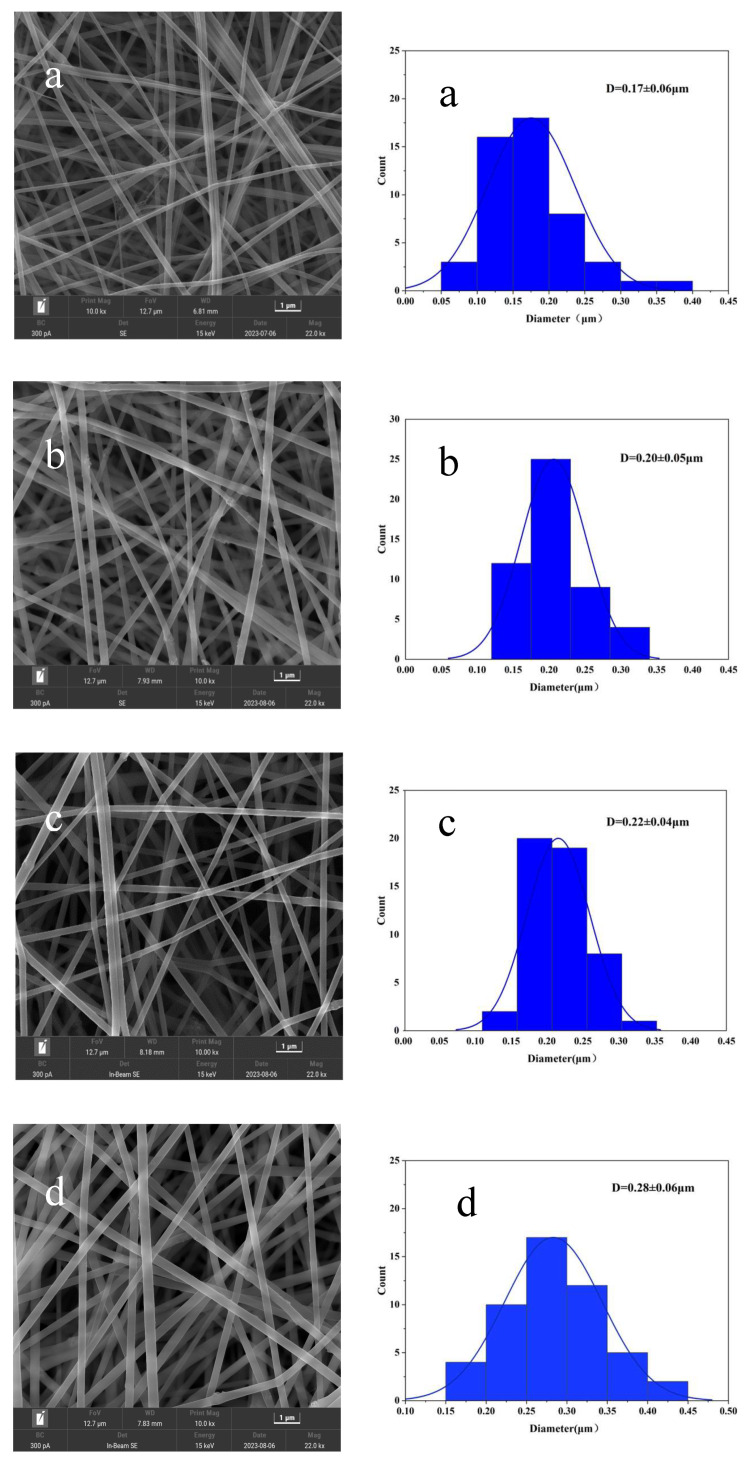
Morphological structure and fiber diameter statistics of the GA/PPA (**a**), GA/PPA/SA I (**b**), GA/PPA/SA II (**c**), GA/PPA/SA III (**d**) nanofibers.

Scanning electron microscopy was utilized to examine the morphological changes of GA nanofibers with the variation of SA concentration. Figure 3 presents the morphological structure and statistical diameter distribution of the nanofibers. The obtained nanofibers exhibited a cylindrical morphology and a porous structure. No significant variation was discovered in the morphological structure of the obtained nanofibers with increasing SA content, except for a small increase in diameter. The average diameters of both GA/PPA nanofibers and GA/PPA/SA nanofibers ranged from 0.17 to 0.28 μm. The average diameter of the GA/PPA nanofibers was (0.17 ± 0.06) μm, while the incorporation of SA resulted in average fiber diameters of (0.20 ± 0.05) μm (GA/PPA/SA I), (0.22 ± 0.04) μm (GA/PPA/SA II), and (0.28 ± 0.06) μm (GA/PPA/SA III), respectively. As depicted in Figure 3, the increase in SA content improved the viscosity of the electrospun solution. The hydrogen bond generated between SA and GA increased the viscosity of the electrospun solution, resulting in an increase in the nanofibers’ diameter [24]. Ali et al. [29] demonstrated that polyphenols act as natural crosslinking agents for GA and can increase the diameter of electrospun nanofibers. 

### 3.4. FT-IR Analysis of Nanofibers

FT-IR spectra showed molecular interactions among all ingredients in the GA/PPA/SA nanofibers. In the GA/PPA nanofibers, as shown in Figure 2b, the peaks at 1643 cm^−1^ and 1543 cm^−1^ were respectively put down to C=O stretching (amide I) and N-H bending (amide II) [18]. In the GA/PPA/SA nanofibers, an aromatic ring stretching peak was observed at 1600 cm^−1^, and an aromatic ring C–H deformation peak was observed at 1074 cm^−1^ [30]. In addition, a prominent peak between 3600 cm^−1^ and 3100 cm^−1^ was observed in all samples, which corresponded to the characteristic gelatin peak for N-H and O-H stretching vibrations [31]. Moreover, the addition of SA may have resulted in the widening of the peak between 3600 cm^−1^ and 3100 cm^−1^, suggesting a decrease in the free N-H and O-H stretching vibrations due to interactions between phenolic acid molecules and gelatin chains [32]. Furthermore, there were notable peaks in the 1540–1175 cm^−1^ region, which corresponded to variants of O-H, C=O, C-H, and C=C, and phenol vibrations [33,34]. The vibrations in the 1175–940 cm^−1^ range were due to stretches of C=C and C=O in the carbohydrate structure and C=O in phenol [30]. As seen in Figure 2b, the Amid I band at 1643 cm−1 for gelatin shifted to a lower wavenumber. Similar redshifts were observed for complex membranes [35] and blended films [36]. The change implies the formation of strong intermolecular interactions including hydrogen bonding and electrostatic attractions between sodium alginate and gelatin chains, with the self-organization of polyelectrolyte complexes.

### 3.5. XRD Analysis of Nanofibers

X-ray diffraction analysis was utilized to inquire into the crystal structure and assess the compatibility of every composition in the nanofibers [37]. Figure 2c illustrates the XRD patterns of the nanofibers. Incorporating SA into the GA/PPA nanofibers did not significantly alter the XRD pattern. However, it slightly heightened the peak intensity of GA/PPA/SA nanofibers at 2θ = 20–22°, which may have been due to the low concentration of SA in the GA/PPA matrix.

### 3.6. TG Analysis of Nanofibers

TGA was applied to assess the thermal stability of nanofibers according to weight loss. The TGA curves of GA/PPA nanofibers with varying weight ratios of SA are shown in Figure 2d. The curves exhibited two primary stages of weight loss between 30 and 600 °C. The first stage occurred from 30 to 150 °C and was put down to the evaporation of free water and bound water [5]. The second stage at 250–600 °C was mainly put down to thermal degradation of gelatin, as previously demonstrated by Kasitnun et al. [7]. Notably, the addition of SA led to increased weight loss at higher temperatures, indicating a reduction in thermal stability due to the presence of SA. A previous study suggested that moderate amounts of SA were able to strengthen the thermal stability of nanofibers through the effect of intermolecular hydrogen bonds, but excess SA hindered the rearrangement of polymeric chains during fiber formation, thus transforming the original compact structure of the GA/PPA/SA matrix [38]. As shown in Figure 2e, a broad peak was detected at approximately 310 °C, indicating thermal decomposition of sugar in the gelatin [20].

### 3.7. Mechanical Properties of Nanofibers

The ability to resist fracture under tensile stress is a crucial and widely measured property of materials used in structural applications. As depicted in Figure 2f, the tensile strength of the nanofibers was initially enhanced and then decreased with the increase in SA concentration. The GA/PPA/SA II nanofibers exhibited the highest tensile strength at (1.74 ± 0.03) MPa, indicating relatively favorable mechanical properties. Conversely, the GA/PPA/SA III nanofibers exhibited the poorest mechanical strength at (1.04 ± 0.04) MPa. This result could be attributed to high concentrations of SA, as an excessive content of 2% (*w*/*v*) may have altered the internal nanofiber structure, resulting in a decline in tensile strength. Similar phenomena were reported by Jun Liu et al. and Mengxia Duan et al. [39,40]. The results show that a suitable amount of SA can strengthen the mechanical properties of GA/PPA nanofibers. However, excessive amounts of SA can achieve the opposite effect. Therefore, in this study, GA/PPA/SA II was considered to be an ideal sample with favorable mechanical properties.

### 3.8. Antioxidant Activity of Nanofibers

The oxidation reaction can result in the formation of rancidity and off-flavors in packaged food. Therefore, antioxidant activity is a significant property of food packaging material [5]. The antioxidant capacities of the GA/PPA nanofibers and GA/PPA/SA nanofibers are presented in Figure 2g. The GA/PPA nanofibers exhibited the weakest antioxidant activity, with a DPPH free-radical scavenging rate of approximately 14.96%. However, the addition of SA obviously strengthened the antioxidant ability of the nanofibers. For instance, the antioxidant capacity of the GA/PPA/SA III nanofibers was about five times greater than that of the GA nanofibers. The heightened antioxidant activity of GA/PPA/SA nanofibers may have been due to the abundance of phenolic hydroxyl groups in the SA, which are capable of eliminating free radicals. However, increasing the SA content from 1% (*w*/*v*) to 2% (*w*/*v*) did not result in an observable difference in the antioxidant capacity of the nanofibers. This lack of variation may have been due to the interaction within the nanofibers affecting the release of SA [25].

### 3.9. Antimicrobial Activity of Nanofibers

The antimicrobial activity of GA/PPA/SA nanofibers as food packaging materials is of great importance for their application. The results are shown in Figure 2h. For both *E. coli* and *S. aureus*, the GA/PPA nanofibers represented the weakest antimicrobial activity, with inhibition zone diameters of 11.98 ± 0.15 mm and 11.92 ± 0.40 mm, respectively. The incorporation of SA significantly enhanced the antimicrobial properties of the GA/PPA nanofibers, so GA/PPA/SA III showed the highest antimicrobial activity, with inhibition zone diameters of 14.53 ± 0.19 mm and 15.6 ± 0.32 mm for *E. coli* and *S. aureus*, respectively. The inhibition zone of the nanofibers for *S. aureus* was obviously larger than that for *E. coli*. This result was put down to the distinction in the cell wall structures difference of Gram-positive bacteria and Gram-negative bacteria [41]. This observation is consistent with the antioxidant activity of the GA/PPA nanofibers and GA/PPA/SA nanofibers.

The bacteriostatic mechanism of SA involves damaging the cell membrane, increasing membrane permeability, reducing intracellular ATP concentration, and elevating intracellular pH. The inhibitory effect of the GA/PPA/SA nanofibers on bacteria was found to be greater for *S. aureus* compared with *E. coli*. This phenomenon is consistent with the study conducted by Ke Yang et al., who studied the effect of SA incorporation on the antibacterial properties of chitosan nanofibers [16]. The good antibacterial performance of GA/PPA/SA nanofibers indicated that GA/PPA/SA nanofibers have the potential to be antibacterial packaging materials.

### 3.10. Sensitivity of Nanofibers to Ammonia

The spoilage of meat products often leads to the formation of volatile nitrogen compounds, for instance, ammonia, dimethylamine, and trimethylamine. Therefore, ammonia is commonly used to mimic the producing of volatile nitrogen compounds during food spoilage. Table 2 presents the ammonia-sensitivity properties of GA/PPA/SA nanofibers. All the GA/PPA/SA nanofibers displayed a transition from a pale white color to light green and eventually dark green, primarily attributed to the structural alteration of anthocyanin. To evaluate the nanofibers’ sensitivity to ammonium vapor over a 60 min period, the changes in E values were monitored. The observed color change of the nanofibers can mainly be explained by the formation of a phenolic oxygen anion in the PPA molecular structure, resulting from an acid–base reaction between the phenolic hydroxyl group of PPA and the hydroxyl groups of ammonia [42].

As seen Table 3, all the GA/PPA/SA nanofibers exhibited larger △E values compared with the GA/PPA/SA nanofibers, with the GA/PPA/SA II nanofibers demonstrating the highest △E value. This can be attributed to the co-pigment effect of SA on PPA stabilization. However, it is important to note that excessive syringic acid, due to the presence of carboxyl groups, might react with ammonia and impact the binding of ammonia and anthocyanins.

### 3.11. Application of Nanofibers in Monitoring Pork Freshness

Based on the aforementioned findings, the GA/PPA/SA II nanofibers exhibited superior mechanical properties, excellent antioxidant and antimicrobial activity, and most importantly, the highest sensitivity to ammonia. Therefore, this study selected the GA/PPA/SA II nanofibers for monitoring the freshness of pork.

**Figure 4:**
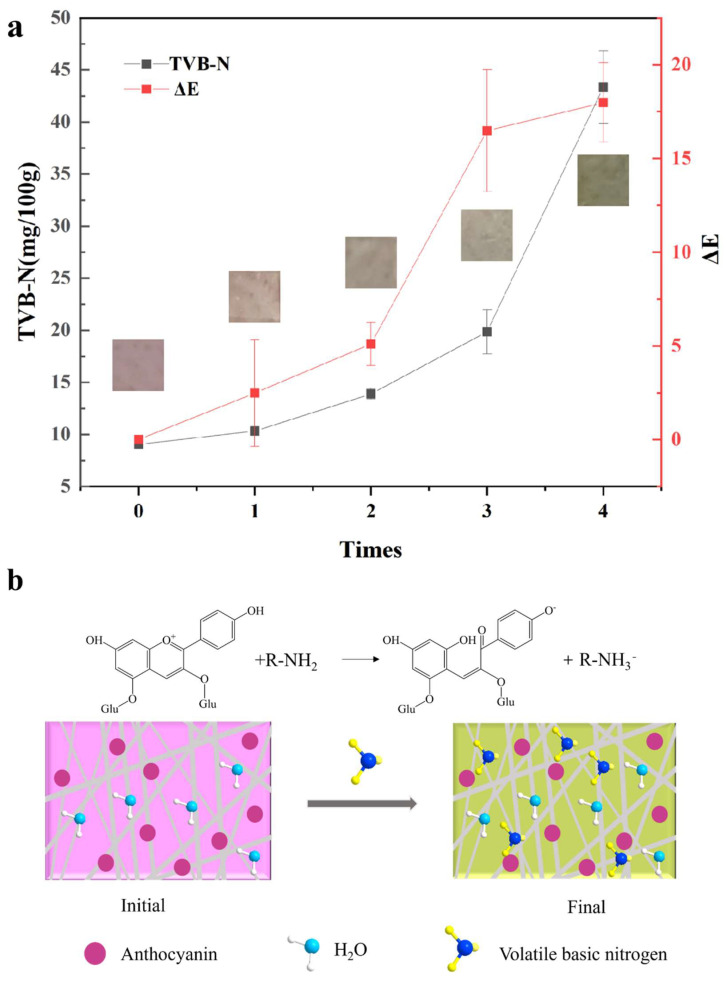
Photographs of GA/PPA/SA II nanofibers when monitoring the freshness of pork at 25 °C for 4 days, the corresponding TVB−N value of the pork, and ΔE value of the nanofibers (**a**), possible mechanism for the color change of the nanofibers under volatile basic nitrogen (**b**).

Meat is a perishable product that changes in its chemical composition during storage due to the growth and reproduction of microorganisms and the catalytic action of endogenous enzymes. Depending on the mechanism of meat spoilage, the degradation of proteins and other nitrogen-containing compounds results in the accumulation of organic amines, generally referred to as TVB-N. TVB-N is frequently utilized as the biomarker for protein and amine degradation [43]. As shown in Figure 4, the GA/PPA/SA II nanofibers initially displayed a dark purple color, which faded by day 2, corresponding to a TVB-N value in the pork sample that approached the reject limitation standard of 15 mg/100 g for livestock and poultry meat (GB 2707-2016 [44]). By days 2–3, the color of the nanofibers continued to lighten, ultimately turning to yellow by day 4. This observable color change indicated the degradation of food due to microbial activity and the release of ammonia vapor as the primary volatile compound [42,45,46].

The GA/PPA/SA II nanofibers initially exhibited a deep purple color, and on day 2, their color showed a ΔE value of 5.10. It has been reported that when the ΔE value of a colorimetric label exceeds 5, the color distinction can be easily discerned by the human eye [47]. On day 3, the TVB-N value reached 19.85 mg/100 g, surpassing the reject limitation set by GB 2707-2016, while the ΔE value increased to 16.48. This indicates that the color change could be readily distinguished by visual observation. In other words, the pork had spoiled and was unsuitable for consumption when the nanofibers turned grey or even green. These findings suggest that the GA/PPA/SA II nanofibers showed the capability to track the freshness of pork over time.

## 4. Conclusions

In this work, we developed multifunctional food packaging nanofibers based on GA, PPA, and SA, via electrospinning technology. The GA/PPA/SA nanofibers exhibited remarkable antioxidant and antibacterial properties. Among all the GA/PPA/SA nanofibers, GA/PPA/SA II displayed the best mechanical properties and ammonia sensitivity. Therefore, this study selected GA/PPA/SA II to evaluate its potentiality for monitoring pork freshness. The color of the GA/PPA/SA II nanofibers changed from dark purple to gray when the TVB-N value reached the rejection limit standard of 15 mg/100 g. The above results highlight the significant potentiality of GA/PPA/SA nanofibers for monitoring the freshness of livestock and poultry meat products.

## Figures and Tables

**Table 1:** Main properties of electrospun solutions.

Solution	GA (g)	PPA (g)	SA (g)	Water (mL)	Acetic Acid (mL)
GA/PPA	7.50	1.20	0.00	12.00	18.00
GA/PPA/SA I	7.50	1.20	0.30	12.00	18.00
GA/PPA/SA II	7.50	1.20	0.45	12.00	18.00
GA/PPA/SA III	7.50	1.20	0.60	12.00	18.00

**Table 2:** Color change of GAPPA and GA/PPA/SA nanofibers in ammonia atmosphere for 60 min.

	0 min	10 min	20 min	30 min	40 min	50 min	60 min
GA/PPA	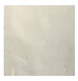	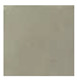	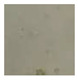	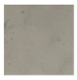	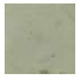	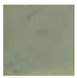	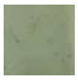
GA/PPA/SAI	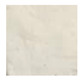	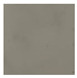	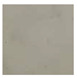	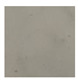	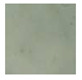	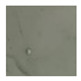	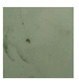
GA/PPA/SAII	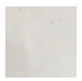	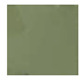	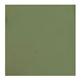	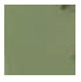	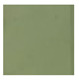	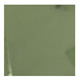	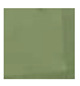
GA/PPA/SAIII	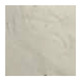	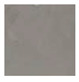	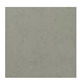	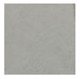	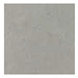	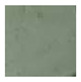	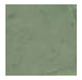

**Table 3:** E values of GA/PPA and GA/PPA/SA nanofibers in ammonia atmosphere for 60 min.

	10 min	20 min	30 min	40 min	50 min	60 min
GA/PPA	10.52 ± 1.29 ^c^	14.72 ± 0.09 ^b^	16.45 ± 1.29 ^ab^	16.46 ± 1.52 ^ab^	17.44 ± 1.12 ^a^	17.97 ± 0.93 ^a^
GA/PPA/SA I	12.63 ± 0.68 ^d^	17.76 ± 1.42 ^c^	18.98 ± 0.49 ^bc^	19.28 ± 0.60 ^b^	21.27 ± 0.37 ^a^	21.66 ± 0.75 ^a^
GA/PPA/SA II	22.84 ± 1.54 ^c^	24.73 ± 0.39 ^b^	26.28 ± 1.15 ^ab^	27.33 ± 0.37 ^a^	27.75 ± 1.14 ^a^	27.97 ± 0.23 ^a^
GA/PPA/SA III	15.67 ± 1.54 ^c^	21.3 ± 0.40 ^b^	23.37 ± 1.15 ^ab^	24.94 ± 0.37 ^ab^	25.41 ± 1.14 ^a^	26.86 ± 0.23 ^a^

Values are given as mean ± SD (*n* = 3). Different letters in the same column indicate significant difference (*p* < 0.05).

## Data Availability

The original contributions presented in the study are included in the article. Further inquiries can be directed to the corresponding author.
